# Anti-nerve growth factor antibody attenuates chronic morphine treatment-induced tolerance in the rat

**DOI:** 10.1186/s12871-016-0242-x

**Published:** 2016-09-05

**Authors:** Bopaiah P. Cheppudira, Alex V. Trevino, Lawrence N. Petz, Robert J. Christy, John L. Clifford

**Affiliations:** 1 Burn Injuries Task Area, United States Army Institute of Surgical Research, 3698 Chambers Pass, San Antonio Military Medical Center, Fort Sam Houston, San Antonio, Texas 78234 USA; 2 Department of Clinical Investigation, United States Army Institute of Surgical Research, 3698 Chambers Pass, San Antonio Military Medical Center, Fort Sam Houston, San Antonio, Texas 78234 USA

**Keywords:** Nerve growth factor, Anti-NGF, Morphine, Tolerance, Hargreaves’ test

## Abstract

**Background:**

Nerve growth factor (NGF) is known to induce inflammation and pain; however its role in opioid-induced tolerance has not been studied. This study investigated the effects of an anti-NGF neutralizing antibody on the development of tolerance following chronic morphine treatment in naïve rats.

**Methods:**

Four groups of rats were used in this study; one treated with saline alone, one with 10 mg/kg of morphine, one with 10 μg of anti-NGF and the other with 10 mg/kg of morphine + 10 μg of anti-NGF, twice per day for 5 days. The route of treatment was subcutaneous (S.C.) for morphine and saline, and intraperitoneal (i.p.) for anti-NGF. Response to a noxious thermal stimulus during the course of drug treatment was assessed (Hargreaves’ test). Further, the change in the NGF levels in the lumbar spinal cord was measured by ELISA.

**Results:**

Our results showed that repeated administration of morphine produced an apparent tolerance which was significantly attenuated by co-administration of anti-NGF (*P* < 0.001). Additionally, the area under the curve (AUC) of the analgesic effect produced by the combination of morphine and anti-NGF was significantly (*P* < 0.001) greater than for saline controls and chronic morphine treated rats. Moreover, the level of NGF in the spinal cord of chronic morphine treated rats was significantly higher (*P* < 0.05) than in both the saline control group and the group receiving simultaneous administration of anti-NGF with morphine. These results indicate that anti-NGF has the potential to attenuate morphine-induced tolerance behavior by attenuating the effects of NGF at the spinal level.

**Conclusion:**

Taken together, our study strongly suggests that the NGF signaling system is a potential novel target for treating opioid-induced tolerance.

## Background

Morphine is a widely used analgesic drug. However, multiple preclinical and clinical studies have shown that chronic administration of morphine is associated with the development of tolerance [1]. Literature shows that opioid-induced tolerance (OIT), defined as a decreased analgesic response following repeated administration of the drug, is a complex phenomenon involving multiple behavioral and cellular adaptations including alterations in a number of pharmacokinetic and pharmacodynamic aspects [2]. Emerging studies have shown that chronic morphine treatment causes release of several inflammatory mediators such as interleukin-1β (IL-1β), interleukin-6 (IL-6), Tumor necrosis factor-α (TNF-α), transforming growth factor-β1 (TGF-β1) and nuclear factor-kappa B (NF-kB) from both neuronal and non-neuronal cells. These inflammatory mediators have been shown to be involved in the development of tolerance [3].

**Fig. 1:**
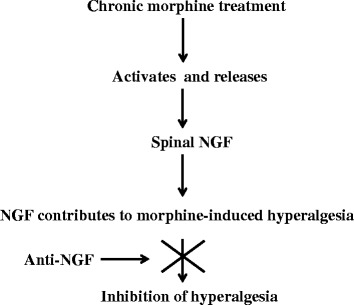
We propose that chronic morphine treatment causes the release of NGF in the spinal cord, which contributes to morphine-induced tolerance. Treatment with a low dose of anti-NGF antibody delays development of tolerance

Nerve growth factor (NGF) is an essential molecule required for the survival of sympathetic and small diameter primary afferent sensory neurons [4]. NGF exerts its biological actions through two receptors: tropomyosin receptor kinase A (trkA) and p75 receptor. There are a substantial number of studies demonstrating involvement of NGF in both central and peripheral nociceptive processing [5]. Elevated levels of NGF have been reported at the peripheral site of injury, in the dorsal root ganglia (DRG) and in the spinal cord of animals with neuropathic pain or/and inflammatory pain [5, 6]. Additionally, sequestration of NGF with antibodies or blockade of NGF receptors with specific inhibitors attenuates allodynia and hyperalgesia [7]. Further, exogenous administration of NGF to healthy animals and human subjects induces dose-dependent allodynia and hyperalgesia [5]. However, the role of NGF in OIT has not been studied. Therefore, we hypothesize that chronic morphine treatment increases spinal cord NGF levels and this contributes to the development of OIT (Fig. 1).

To test this hypothesis we used a morphine-induced tolerance protocol on rats and examined the effects of treatment with NGF neutralizing antibodies on pain behavior and on NGF levels in the spinal cord.

## Methods

### Animals

Male Sprague—Dawley rats (300–320 g) were housed 2/cage under standard conditions (12:12 h light: dark cycle with ad libitum access to food and water). All studies were approved by the U.S. Army Institute of Surgical Research Institutional Animal Care and Use Committee and conform to federal guidelines and guidelines of the International Association for the Study of Pain. This study has been conducted in compliance with the Animal Welfare Act, the implementing Animal Welfare Regulations, and the principles of the Guide for the Care and Use of Laboratory Animals. The animal facility is fully accredited by the Association for the Assessment and Accreditation of Laboratory Animal Care, International (AAALAC, Intl.).

### Drugs and treatments

Anti-NGF-β antibody (lyophilized powder, Sigma-Aldrich, N8773) and morphine sulfate (Hospira Inc.) were dissolved or diluted in sterile phosphate buffered saline to desired concentrations. Four groups of rats (*n* = 6) were used for this study. They were randomly assigned to receive the following treatments: subcutaneous (s.c.) injection of saline (0.5 ml); morphine (10 mg/kg in 0.5 ml, s.c.) along with intraperitoneal (i.p.) saline (0.5 ml); morphine (10 mg/kg in 0/5 ml, s.c) along with anti-NGF (10 μg in 0.5 ml, i.p.); or anti-NGF (10 ug in 0.5 ml, i.p.). The i.p. saline or anti-NGF was administered within 1–2 min after morphine administration. Each regimen was administered twice daily for 5 consecutive days. The time of drug administration was between 9–10 a.m. and 5–6 p.m.

### Assessment of thermal sensitivity

Assessment of response to a noxious thermal stimulus (Hargreaves test) was performed using a plantar analgesia instrument (Model 390; IITC Life Science, Woodland Hills, CA, USA) [8]. Briefly, rats were acclimatized to the behavioral assessment room for 30 min and to the Plexiglas chamber, which was placed above a heated (35 C) transparent glass surface for 30 min before the assessment. The light beam was focused to the mid-plantar surface of the hind paw. The time between the application of thermal stimuli and hind paw withdrawal response was defined as the paw withdrawal latency in sec (PWL). The intensity of the beam was set to 40 % to produce baseline PWL of approximately 10 s in naïve rats. Three trials for each hind paw, with an interval of 5 min, were averaged and for data analysis the scores from both left and right paws were combined to yield the mean PWL of each rat. The percentage of maximum possible antinociceptive effect (% MPAE) was calculated using the formula: % MPAE = (Post-morphine treatment – Pre-morphine treatment) / (20-pre-morphine treatment) × 100. The area under the curve (AUC) of PWL plotted over time was calculated using the trapezoid rule. Additionally, the PWL before treatment (baseline) on each test day (day 1, day 3 and day 5) was analyzed to observe changes in baseline sensitivity to thermal stimuli during the experimental period. The behavioral experimenters were blinded to the treatments.

### Enzyme-linked immunosorbent assay (ELISA)

Following the final behavioral testing on day 5, rats from each experimental group were sacrificed by decapitation and the spinal cord tissue from lumbar sections L4 to L6 was harvested and snap frozen in liquid nitrogen and stored at −80 °C. Quantitative determination of NGF content in tissue samples was performed by ELISA, according to the manufacture’s instruction (Biosensis; catalogue number BEK-2214). Briefly, after weighing, tissue was re-suspended in approximately 100 μl extraction buffer per 10 mg tissue, homogenized for 8–10 min and placed on ice for 30 min. The homogenate was then centrifuged at 100,000 × g and 4 °C for 30 min and the clear supernatant collected into clean tubes. The sample was diluted (1:2) in incubation/neutralization buffer before loading into the wells of an ELISA plate. The NGF protein concentration of the sample was calculated based on a standard NGF concentration-absorbance curve (OD 450 nm) and expressed as pg/mL of protein (Biosensis; catalogue number BEK-2214).

### Statistical analysis

GraphPad Prism 5 statistical software was used to analyze the experimental data. All data were expressed as mean ± SEM. The data obtained from behavioral and ELISA experiments were analyzed using either one-way analysis of variance (ANOVA) or repeated-measures ANOVA (Two-Way ANOVA) followed by Bonferroni’s post-hoc test to compare the difference among groups. The statistical significance was set at a level of *p* < 0.05.

## Results

The development of OIT and effects of anti-NGF treatment on that process was measured using the nociceptive thermal test. A total of 24 rats (*n* = 6/group) were included in the study.

### Repeated morphine treatment alters baseline sensitivity to thermal stimuli

As shown in Table 1, before treatment, the baseline PWL in all groups ranged from 8.1–9.6 s, and ANOVA showed no significant differences between groups (*P* > 0.05). The pre-treatment paw withdrawal latencies of Sal-Sal group did not differ significantly between days of testing (*P* > 0.05). In contrast, the pre-treatment PWL progressively decreased in rats from Mor-Sal, Mor + Anti-NGF and Anti-NGF-Sal groups. However, the significant difference was observed on day 5 compared to corresponding day 1 baseline latency (*P* < 0.05). Additionally, the rats that received Mor-Sal and Mor + Anti-NGF showed significant reduction in before treatment PWL on day 5 (*P* < 0.05) compared to both Sal-Sal and Anti-NGF-Sal groups. This also indicates that the greater reduction in the basal nociceptive behaviors is due to chronic morphine treatments but not because of frequent handling and behavioral testing of animals during the experimental period as the baseline values of PWL did not alter in Sal-Sal-treated group.

**Table 1:** Chronic morphine treatment alters baseline thermal sensitivity

Mean paw withdrawal latencies (sec)			
	Day 1	Day 3	Day 5
Sal-Sal	8.87 ± 0.48	7.708 ± 0.29	8.498 ± 0.32
Mor-Sal	9.518 ± 0.48	7.891 ± 0.44	6.326 ± 0.36 ** ###
Mor + Anti-NGF	8.166 ± 0.42	8.042 ± 0.61	5.709 ± 0.22 ** ###
Anti-NGF-Sal	9.631 ± 0.16	9.049 ± 0.22	8.326 ± 0.15 ***

Data are expressed as mean ± SEM, and refer to paw withdrawal latencies prior to Saline-Saline, Morphine-Saline, Morphine + Anti-NGF or Anti-NGF-Saline treatment on the day 1, day 3 and day 5. ** *P* < 0.01, *** *P* < 0.001 as compared to day 1 pre-treatment (baseline) of each corresponding group. ^###^ *P* < 0.001 represents significant difference compared to day 5 of Sal-Sal group

### Anti-NGF attenuated morphine-induced tolerance

**Fig. 2:**
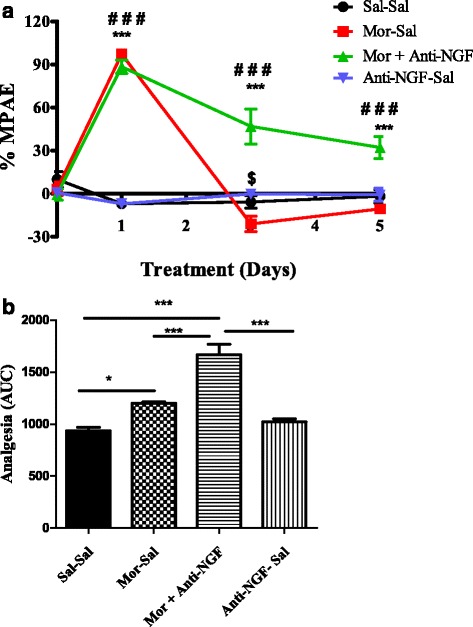
Effects of anti-NGF antibody on the development of morphine-induced tolerance (OIT). **a**. Hind paw withdrawal latency to thermal stimulation expressed as percentage of maximal possible effect (% MPAE) decreased in rats that received chronic morphine treatment (10 mg/kg, s.c.) on days 3 and 5. Co-administration of morphine with anti-NGF (10 μg, i.p.) significantly reduced the decreased withdrawal latency on days 3 and 5. Each data point represents the mean ± S. E. M. of six rats per group. ***P < 0.001 compared with either Morphine-Saline group or Anti-NGF-Saline; ###*P* < 0.001 compared Saline-Saline group or Anti-NGF-Saline group; $ *P* < 0.05 compared to Morphine-Saline group. **b**. Area under the curve (AUC) from 0 to 120 h, *P < 0.01 compared to Saline-Saline group, ***P < 0.0001 compared to Saline-Saline or Anti-NGF-Saline and Morphine-Saline groups

A single injection of morphine or morphine + anti-NGF significantly increased the % MPAE in comparison to the saline-treated group (*P* < 0.001) (Fig. 2). Morphine treatment alone resulted in a 97.12 % MPAE whereas the effect of the morphine + anti-NGF combination produced 88.45 % MPAE. On day 1, there was no significant difference in the effect between these two treatment groups (*P* > 0.05). Rats receiving repeated morphine administration had a considerably reduced pain threshold by day 3 and this continued until day 5 of treatment, indicating development of tolerance. These chronic morphine treated rats showed a slightly lower % MPAE than the saline control group on days 3 & 5, but the effect was not statistically significant (*P* > 0.05). In contrast, simultaneous administration of anti-NGF with morphine increased the % MPAE on days 3 and 5, to 67.95 and 42.71, respectively. These % MPAEs were significantly higher than for the chronic morphine and saline control rats (*P* < 0.001), suggesting a potential effect of anti-NGF in blocking chronic morphine-induced tolerance. The anti-NGF effect on day 5 was approximately 25 % less than on day 3. Treatment with anti-NGF alone did not alter the paw withdrawal latency and the nociceptive threshold was almost same as the saline control group. However, on day 3 chronic morphine treated rats showed a 20.94 % lower MPAE than anti-NGF alone treated rats (*P* < 0.05).

As shown in Fig. 2b, when the analgesic activity of morphine and morphine + anti-NGF was expressed as the AUC for PWL plotted against time (AUC _0–120_ h), the value for the morphine treated group was 266 units higher than for the saline-treated control group (*P* < 0.05), and the value for the morphine + anti-NGF group was 732 units higher than for the saline controls (*P* < 0.001) and 645 units more than for the anti-NGF alone treatment group (*P* < 0.001).

### Repeated administration of morphine increases spinal cord NGF levels

**Fig. 3:**
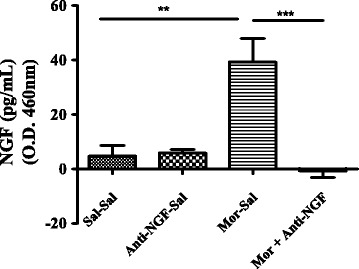
Chronic morphine treatment increases the levels of NGF in the spinal cord as measured by ELISA (Fig. 3). The quantity of NGF protein in the lumbar sections (L4-L6) of chronic morphine treated group showed more than three-fold higher than either saline alone or anti-NGF alone treated groups. Co-administration of anti-NGF antibody significantly decreased the NGF content. No significant difference among saline, Anti-NGF and morphine + anti-NGF-treated groups was observed. Data are expressed as mean ± SEM. ** *P* < 0.001 compared to Saline-Saline group and ## *P* < 0.001compared to Morphine + anti-NGF group

As illustrated in Fig. 3, the NGF protein expression in the lumbar region of the spinal cord (L4-L6) of chronic morphine treated rats was significantly higher than in saline treated rats (*P* < 0.001). Rats that received repeated administration of morphine + anti-NGF expressed significantly lower NGF levels in comparison to chronic morphine treated rats (*P* < 0.001) but with levels similar to the saline control group (*P* > 0.05). Repeated administration of anti-NGF alone resulted in significantly lower amounts of NGF in comparison to the morphine alone group (*P* < 0.01), and those levels were similar to saline control and the morphine + anti-NGF treated group.

## Discussion

Currently, morphine is extensively used as a potent analgesic drug for many types of moderate to severe, acute and chronic pain conditions. However, prolonged use of morphine induces tolerance, thus reducing its clinical utility [3, 9, 10]. OIT mechanisms are complex. In animal models, a decrease in analgesic response to noxious stimuli following short- and/or long-term exposure to opioids is characterized as tolerant behavior [11, 12]. In agreement with these reports, we also demonstrated the development of tolerance in naïve rats to thermal stimuli following chronic administration of morphine. The analgesic effect of morphine, as indicated by increased %MPAE (increased PWL), was significantly reduced by day 3 and this effect continued until the end of the experiment.

There is ample evidence demonstrating that anti-NGF attenuates pain hypersensitivity in a wide variety of animal pain models [4, 5, 8, 13, 14] and it was shown to be effective through multiple routes of administration: intraperitoneal (i.p.), subcutaneous (s.c.), intrathecal and local application at the site of injury [13–15]. Anti-NGF acts by binding and neutralizing the action of NGF on nociceptors. Until the present study, the effect of anti-NGF on morphine-mediated tolerance had not been determined. We demonstrate that simultaneous i.p. administration of a low dose of anti-NGF can attenuate the decreased % MPAE of morphine that develops after multiple morphine administrations. However, the analgesic effect of morphine was not fully restored and also there is a reduced attenuation of tolerance on day 5 compared to day 3. It is possible that, in addition to NGF, other neurotrophins such as brain-derived neurotrophic factor (BDNF) and neurotrophin-3 (NT-3) could also contribute to opioid tolerance. NGF, BDNF and NT-3 all bind to the neurotrophin receptor p75 (p75 NTR) in addition to their cognate tyrosine kinase receptors – TrkA, TrkB, and TrkC. Interestingly, a recent study has shown attenuation of opioid analgesic tolerance in p75NTR null mice [16]. Likewise, upregulation of BDNF and NT-3 occurs in the brain regions that are involved in opioid dependence and withdrawal following repeated morphine treatment [17–19]. These studies support the likely role for BDNF and NT-3, in addition to NGF, in opioid tolerance mechanisms. Moreover, other mechanisms are expected to contribute to opioid-induced tolerance, apart from those involving neurotrophins. For example, previous studies have shown that glutamate signaling through NMDA receptors [20], glial cell activation [3], nitric oxide signaling [21] and down-regulation of opioid receptor expression [22], following repeated opioid treatment all can greatly decrease the pain threshold. Further, the AUC of the analgesic effect of the morphine + anti-NGF combination was significantly greater than for the morphine alone group and for the saline controls, supporting the hypothesis that NGF signaling mediates OIT. Additionally, anti-NGF treatment alone had no effect on the nociceptive threshold in naïve rats, indicating that the anti-hyperalgesic effect of anti-NGF in morphine tolerant rats is likely due to neutralizing the effects of elevated NGF, and not to some other anti-nociceptive effect of anti-NGF.

It has been reported that morphine acts on neuronal and glial cells to cause the release of a number of inflammatory mediators that are involved in the development of OIT [3]. Our ELISA results show that chronic morphine treatment also induces the release of NGF in the spinal cord and excessive expression of NGF correlates with decreased analgesic behavior in tolerant animals. Earlier studies have shown that nerve injury, inflammation and tissue injury elevates NGF levels at the site of injury, dorsal root ganglia and spinal cord [14]; however, our data for the first time shows a morphine-mediated increase of NGF levels in a non-injured condition. It is also possible that subcutaneous, chronic administration of morphine could increase NGF at the peripheral sites, and that this could contributes to tolerance. Similarly, intraperitoneal injections of anti-NGF could also block NGF action at the periphery, in addition to its effects at the spinal cord level. However, this study only assessed the NGF levels in the spinal cord in morphine tolerant rats. Additionally, NGF mediated hyperalgesia has been attributed to inflammation, degranulation of mast cells, activation of sympathetic nerve terminals and action on TrKA-expressing primary sensory neurons [5, 7, 13, 14]. Further studies are needed to determine whether similar mechanisms are responsible for the effects of NGF signaling in mediating OIT.

There is a commonly held concern regarding side effects of high dose antibodies as therapeutics. The anti-NGF dose that we used in this study is relatively low (10 μg/injection) compared to amounts used in other antibody therapy studies [13, 23]. Even so, less than this dose was shown to be effective in attenuating mechanical hyperalgesia in an inflammatory pain and spinal cord injury models [15, 23]. We propose that higher doses of anti-NGF could be needed toabrogate OIT or opioid-induced hypersensitivity in animal models and in the clinic. We also propose that the route of administration will likely be an important factor in effectiveness.

The present study showed that the development of OIT can be attenuated by co-administration of a low dose of anti-NGF, along with morphine. Moreover, chronic morphine treatment caused increased expression of NGF in the lumbar (L4-L6) region of the spinal cord and this was significantly reduced to near control levels in the rats receiving repeated morphine + anti-NGF. These results strongly suggest that NGF signaling plays a role in morphine-induced reduction of analgesic effects and that suppression of NGF action with a neutralizing antibody reduces OIT. This reduction is the result of a true attenuation of tolerance as anti-NGF treatment alone did not alter the baseline levels of NGF.

## Conclusions

Taken together, our study strongly suggests that a low dose of anti-NGF neutralizing antibody can reduce OIT by blocking the action of NGF (Fig. 1). The NGF signaling system is a potential novel target for treating OIT.

## Abbreviations

BDNF: Brain derived neurotropic factor
DRG: Dorsal root ganglia
IL-1β: Interleukin-1β
IL-6: Interleukin-6
NF-kB: Nuclear factor-kappa B
NGF: Nerve growth factor
NT-3: Neurotrophin-3
OIT: Opioid-induced tolerance
TGF-β1: Transforming growth factor-β1
TNF-α: Tumor necrosis factor-α
TrkA: Tropomyosin receptor kinase A
